# Association of a PAI-1 Gene Polymorphism and Early Life Infections with Asthma Risk, Exacerbations, and Reduced Lung Function

**DOI:** 10.1371/journal.pone.0157848

**Published:** 2016-08-24

**Authors:** Seong H. Cho, Jin-Young Min, Dong Young Kim, Sam S. Oh, Dara R. Torgerson, Maria Pino-Yanes, Donglei Hu, Saunak Sen, Scott Huntsman, Celeste Eng, Harold J. Farber, William Rodriguez-Cintron, Jose R. Rodriguez-Santana, Denise Serebrisky, Shannon M. Thyne, Luisa N. Borrell, L. Keoki Williams, William DuPont, Max A. Seibold, Esteban G. Burchard, Pedro C. Avila, Rajesh Kumar

**Affiliations:** 1 Division of Allergy-Immunology, Department of Medicine, Northwestern University, Chicago, Illinois, United States of America; 2 Division of Allergy-Immunology, Department of Internal Medicine, University of South Florida, Tampa, Florida, United States of America; 3 Department of Otolaryngology, Northwestern University, Chicago, Illinois, United States of America; 4 Department of Medicine, University of California, San Francisco, California, United States of America; 5 Division of Biostatistics, Department of Preventive Medicine, UTHSC, Memphis, Tennessee, United States of America; 6 Department of Pediatrics, Section of Pulmonology, Baylor College of Medicine, Texas Children's Hospital, Houston, Texas, United States of America; 7 Veterans Caribbean Health Care System, San Juan, Puerto Rico, United States of America; 8 Centro de Neumologia Pediatrica, CSP, San Juan, Puerto Rico, United States of America; 9 Pediatric Pulmonary Division, Jacobi Medical Center, Bronx, New York, United States of America; 10 Department of Pediatrics, University of California, San Francisco, California, United States of America; 11 Department of Health Sciences, Lehman College, CUNY, New York, New York, United States of America; 12 Department of Internal Medicine, Henry Ford Health System, Detroit, Michigan, United States of America; 13 Center for Health Policy and Health Services Research, Henry Ford Health System, Detroit, Michigan, United States of America; 14 Department of Biostatistics, Vanderbilt University Medical School, Nashville, Tennessee, United States of America; 15 Center for Genes, Environment and Health, National Jewish Health, Denver, Colorado, United States of America; 16 Division of Allergy-Immunology, Department of Pediatrics, Northwestern University, Chicago, Illinois, United States of America; 17 The Ann and Robert H. Lurie Children’s Hospital of Chicago, Chicago, Illinois, United States of America; National Heart and Lung Institute, UNITED KINGDOM

## Abstract

**Background:**

*Plasminogen activator inhibitor-1* (*PAI-1*) is induced in airways by virus and may mediate asthmatic airway remodeling. We sought to evaluate if genetic variants and early life lower respiratory infections jointly affect asthma risk.

**Methods:**

We included Latino children, adolescents, and young adults aged 8–21 years (1736 subjects with physician-diagnosed asthma and 1747 healthy controls) from five U.S. centers and Puerto Rico after excluding subjects with incomplete clinical or genetic data. We evaluated the independent and joint effects of a *PAI-1* gain of function polymorphism and bronchiolitis / Respiratory Syncytial Virus (RSV) or other lower respiratory infections (LRI) within the first 2 years of life on asthma risk, asthma exacerbations and lung function.

**Results:**

RSV infection (OR 9.9, 95%CI 4.9–20.2) and other LRI (OR 9.1, 95%CI 7.2–11.5) were independently associated with asthma, but *PAI-1* genotype was not. There were joint effects on asthma risk for both genotype-RSV (OR 17.7, 95% CI 6.3–50.2) and genotype-LRI (OR 11.7, 95% CI 8.8–16.4). A joint effect of genotype-RSV resulted in a 3.1-fold increased risk for recurrent asthma hospitalizations. In genotype-respiratory infection joint effect analysis, FEV_1_% predicted and FEV_1_/FVC % predicted were further reduced in the genotype-LRI group (β -2.1, 95% CI -4.0 to -0.2; β -2.0, 95% CI -3.1 to -0.8 respectively). Similarly, lower FEV_1_% predicted was noted in genotype-RSV group (β -3.1, 95% CI -6.1 to -0.2) with a trend for lower FEV_1_/FVC % predicted.

**Conclusions:**

A genetic variant of *PAI-1* together with early life LRI such as RSV bronchiolitis is associated with an increased risk of asthma, morbidity, and reduced lung function in this Latino population.

## Introduction

Asthma affects more than 25 million people in the United States including 9.3% of all US children, with $56 billion in annual healthcare and indirect costs.[1] Studies have suggested a range of 47 to 95% heritability for asthma,[2–4] with multiple associated genetic variants.[5] However, the individual effects of these variants are small,[6,7] leading to questions about whether these genetic influences are more relevant in the broader context of specific environmental exposures.[8] Among the early environmental exposures associated with asthma, viral respiratory infections are among the most important.[9,10] While interaction between viral illness and genetic variants on asthma risk has been reported,[11] mechanisms remain unclear.

A gene-infection interaction promoting airway remodeling and lung function decline may be important both in asthma and generating severe asthma phenotypes. The plasmin and fibrinolytic pathway may be particularly relevant in airway remodeling and upper respiratory infection is associated with increased fibrinogenic activities in subjects with recurrent wheezing or asthma.[12,13] Specifically, *plasminogen activator inhibitor-1* (*PAI-1*) promotes fibrosis,[14,15] and blocking of this enzyme prevents extracellular matrix (ECM) deposition.[16,17] URI increases airway *PAI-1* levels, with virus inducing *PAI-1* production in human airway epithelial cells.[12] *PAI-1* promoter site genetic variants are strongly associated not only with plasma *PAI-1* levels,[18] but also with increased risk of asthma, decreased forced expiratory volume in 1 second (FEV_1_), and airway hyperreactivity.[19] [20]

We utilized the Genes-environments and Admixture in Latino Americans (GALA II) study to test our hypothesis that a *PAI-1* polymorphism in combination with infection in early life may be associated with asthma, asthma severity, and worse lung function.

## Materials and Methods

### Recruitment

Latino children, adolescents, and young adults aged 8–21 years from five centers (Chicago, Illinois; Bronx, New York; Houston, Texas; San Francisco Bay Area, California; and Puerto Rico) were enrolled in the GALA II cohort study from 2006 to 2011 (n = 4157 children of whom 2022 had asthma and 2135 were healthy controls). We excluded subjects who had incomplete genetic or clinical data for relevant covariates (n = 286 asthmatics and 388 controls), yielding an analyzing sample size of 3483 subjects (1736 with asthma and 1747 without asthma). Further details are available on the online supporting information file.

### PAI-1 genotyping for *rs2227631*

Genome-wide genotyping was performed with the Axiom LAT1 array (World Array 4; Affymetrix, Santa Clara, Calif) as previously described.[21] The *A* allele of the promoter site SNP, *rs2227631*, for the PAI-1 gene is a gain of function mutation associated with higher plasma levels of *PAI-1*,[18] and is included in this chip. In initial exploratory analyses, we evaluated the individual effects of *AG* and *AA* genotypes in combination with infection on asthma risk. While there was a dose effect, numbers were not sufficiently large to separately analyze the *AA*-infection group (for example *AA*-RSV is only 0.9% (31/3446). The *AG* and *AA* groups were combined for the primary analysis. For completeness, a secondary analysis for the primary outcome of asthma is presented in the E-tables with genotype expressed as *GG*, *AG*, and *AA*.

### Bronchiolitis / RSV

Symptomatic bronchiolitis / RSV episodes requiring medical attention within the first 2 years of life was ascertained by the following question: “Was <CHILD> diagnosed with bronchiolitis or RSV before age 2 yrs.”

### Other lower respiratory illness

Symptomatic lower respiratory tract illnesses (LRI) requiring medical attention within the first 2 years of life was ascertained by the following question: “Was <CHILD> seen by a doctor for chest illness before age 2 yrs.”

### Subject exposure classification

Subjects were divided into subgroups to identify the independent and joint effects of LRI requiring medical attention and *rs2227631* genotype as follows: *GG* without LRI (*GG*-No LRI), *AG/AA* without any history of LRI (*AG/AA* No LRI), GG with history of LRI (*GG*+LRI), and *AG/AA* with history of LRI (*AG/AA*+LRI). A similar grouping was carried out for genotype and symptomatic bronchiolitis / RSV, with “no RSV” indicating that there was no severe symptomatic RSV bronchiolitis requiring medical attention (i.e. no severe RSV).

### Outcome measures

The primary outcome was report of physician diagnosed asthma. Secondary outcomes included asthma exacerbations and lung function. Spirometry was performed using a KoKo spirometer (nSpire Health, Longmont, CO) according to the guidelines of the American Thoracic Society/European Respiratory Society. Measures analyzed included percent predicted FEV1, FVC, and FEV1/FVC ratio (National Health and Nutrition Examination Survey III reference standards). Subjects with asthma were categorized for exacerbation status based on each the following criteria assessed over the previous 12 months: oral steroid use for 2 or more weeks, ≥ 2 emergency room (ER) visits, and ≥ 2 hospital admissions.

### Replication cohort

The Study of African Americans, Asthma, Genes & Environments II (SAGE II) was used to replicate the associations found in the GALA II study and is described elsewhere.[22] Briefly, the SAGE II study is an ongoing clinic-based multicenter asthma case-control study, including African American subjects with asthma (n = 666) recruited from the San Francisco bay area, conducted in parallel to GALA II using identical protocols and questionnaires.

### Statistical analysis

For descriptive statistics, the study population was divided into asthma and control subjects, and χ^2^ tests and *t* tests performed to describe differences in terms of demographic and clinical characteristics. Analyses on the outcome of asthma diagnosis included all subjects. However, asthma exacerbations and lung function analyses were limited to subjects with asthma. Separate logistic regression analyses were performed to estimate the associations of asthma with the *PAI-1* genotype at the *rs2227631* locus, early-infection (RSV or LRI) history, and the joint effect of genotype and infection. Similar logistic regression analyses were carried out for the outcome of asthma exacerbations limited to subjects with asthma. Both analyses controlled for age, sex, ethnicity, ancestry, socioeconomic factors, environmental factors (history of farm animal exposure, received antibiotics during first year), family history of asthma, African and European genetic ancestry, and recruitment site. For the analysis of lung function (percent predicted values) within asthmatics we used a multivariate linear regression model, adjusting for age, sex, ethnicity, socioeconomic factors, environmental factors (history of smoke exposure), medication for asthma control, and recruitment site. Lung function analyses which included the SNP or SNP-infection analyses also controlled for ancestry. For the asthma analyses, separate models were also generated to test for the significance of interaction terms for infection and risk genotype, including terms for the main effects of genotype risk allele and infection history.

Replication of these analyses was carried out in the SAGE study using the same approach. All statistical analyses were performed using SPSS software (Version 22.0, Statistical Package for Social Science, Chicago, IL, USA). P values < 0.05 were considered statistically significant.

This study was approved by the institutional review boards at each study center (IRB for USCF, IRB for Northwestern University, IRB for The Ann and Robert H. Lurie Children’s Hospital of Chicago, IRB for IRB for Texas Children’s Hospital Baylor College of Medicine, IRB for Veteran’s Caribbean Health Care System, IRB for Centro Neumologica Pediatrica, IRB for Jacobi Medical Center, IRB for CUNY). Written informed consent was obtained from the parents or legal guardians of all children and adult participants, and written informed assent was obtained from all children aged 12–18 years.

Data used in the performance of this analysis is included with the paper in S1 File.

## Results

### Baseline characteristics

Table 1 presents the distribution of selected characteristics for the overall study sample divided by asthma status. The mean age of the study population was 13 years and majority of participants were Mexicans (36.5%) or Puerto Rican (42.6%). Compared to those without asthma, subjects with asthma were more likely to have had a symptomatic episode of RSV bronchiolitis or other symptomatic lower respiratory illness before age 2 years old (9.3% vs 0.9%; or 55.4% vs 10.1%, respectively), but there was no significant difference in PAI-1 genotype. Other differences by asthma status were minor in magnitude and are described in the online supplement.

**Table 1:** Demographic and clinical characteristics of subjects.

Variable	Total (n = 3483)	Asthma (n = 1736)	Non-asthma (n = 1747)	*P* value
Age, mean (SD), years	13.18 (3.49)	12.60 (3.32)	13.75 (3.56)	**0.000** ^a^
Male, No (%)	1735 (49.8)	961 (55.4)	774 (44.3)	**0.000** ^b^
BMI, mean (SD)	23.51 (6.66)	23.25 (6.56)	24.60 (6.93)	**0.000** ^a^
Ethnicity, No (%)				
Mexican	1271 (36.5)	602 (34.7)	669 (38.3)	**0.027** ^b^
Puerto Rican	1485 (42.6)	735 (42.3)	750 (42.9)	0.724
Other Latino	620 (17.8)	338 (19.5)	282 (16.1)	**0.010** ^b^
Mixed Latino	106 (3.0)	61 (3.5)	45 (2.6)	0.107
Ancestry proportion, mean (SD)				
African	0.14 (0.13)	0.15 (0.13)	0.13 (0.12)	**0.000** ^a^
European	0.59 (3.81)	0.54 (0.19)	0.65 (5.37)	0.216
Country born: USA, No (%)	1703 (49.6)	732 (43.0)	971 (56.2)	**0.000** ^b^
Recruited center, No (%)				
IL	639 (18.4)	310 (17.9)	329 (18.8)	0.492
TX	365 (10.5)	197 (11.4)	168 (9.6)	0.087
NY	545 (15.7)	290 (16.8)	255 (14.6)	0.078
SF	636 (18.3)	317 (18.3)	319 (18.3)	0.955
PR	1289 (37.1)	614 (35.5)	675 (38.7)	0.056
Frequency of allergic sensitization	1809 (52)	1076 (62)	733 (42)	
Family history of Asthma, No (%)				
Mother	771 (22.8)	561 (33.1)	210 (12.4)	**0.000** ^b^
Father	463 (14.4)	334 (20.8)	129 (8.0)	**0.000** ^b^
Siblings	1343 (41.6)	866 (53.4)	477 (29.8)	**0.000** ^b^
Infection history before age 2 yrs, No (%)				
RSV bronchiolitis	177 (5.1)	161 (9.3)	16 (0.9)	**0.000** ^b^
LRI	1126 (32.7)	952 (55.4)	174 (10.1)	**0.000** ^b^
Environments				
Pet exposure during 1^st^ yr of life, No (%)				
Cat	371 (10.8)	179 (10.4)	192 (11.1)	0.501
Dog	1165 (33.8)	542 (31.4)	623 (36.1)	**0.003** ^b^
Farm animals	358 (10.4)	148 (8.6)	210 (12.1)	**0.001** ^b^
Smoke, No (%)				
Mother smoke during pregnancy	168 (4.9)	97 (5.6)	71 (4.1)	**0.039** ^b^
Adults smoke before age 2yrs	784 (25.3)	437 (27.7)	347 (22.8)	**0.002** ^b^
Children ever tried smoking	150 (4.3)	58 (3.3)	92 (5.3)	**0.005** ^b^
Children current smoking	1 (0.1)	0 (0.0)	1 (0.2)	0.450
Received antibiotics during 1^st^ yr of life, No (%)	1288 (41.7)	774 (49.7)	514 (33.6)	**0.000** ^b^
Daycare, No (%)	812 (23.8)	443 (25.9)	369 (21.7)	**0.004** ^b^
Socioeconomic characteristics				
Mother education, No (%)				
< High school	1356 (38.9)	656 (37.8)	700 (40.1)	0.168
High school	913 (26.2)	471 (27.1)	442 (25.3)	0.219
Some college	1214 (34.9)	609 (35.1)	605 (34.6)	0.781
Income, No (%)				
< $25,000	1227 (35.2)	623 (35.9)	604 (34.6)	0.417
$25–75,000	1195 (34.3)	634 (36.5)	561 (32.1)	**0.006** ^b^
> $75,000	1061 (30.5)	479 (27.6)	582 (33.3)	**0.000** ^b^
Insurance, No (%)	2195 (93.1)	1631 (95.2)	1564 (91.0)	**0.000** ^b^
rs2227631, No (%)				
GG	1405 (40.3)	676 (38.9)	729 (41.7)	0.093
AG	1583 (45.4)	801 (46.2)	782 (44.8)	0.414
AA	495 (14.3)	259 (14.9)	236 (13.5)	0.233
AG+AA	2078 (59.7)	1060 (61.1)	1018 (58.3)	0.093
Lung function, mean (SD)				
FEV_1_% predicted (pre-BD)	92.45 (15.65)	91.03 (16.03)	98.65 (12.04)	**0.000** ^a^
FVC % predicted (pre-BD)	96.03 (15.62)	95.55 (16.17)	98.14 (12.78)	**0.000** ^a^
FEV_1_/FVC ratio % predicted (pre-BD)	96.53 (8.86)	95.54 (9.00)	100.86 (6.70)	**0.000** ^a^

BMI, body mass index; IL, Illinois; TX, Texas; NY, New York; SF, San Francisco; PR, Puerto Rico; RSV, respiratory syncytial virus; FEV1, forced expiratory volume in 1 second; FVC, forced vital capacity; BD, bronchodilator

^a^ *P* < 0.05 from the *t* test for Asthma vs Non–Asthma.

^b^ *P* < 0.05 from the χ2 test for Asthma vs Non–Asthma.

### Risk of asthma

**Fig 1:**
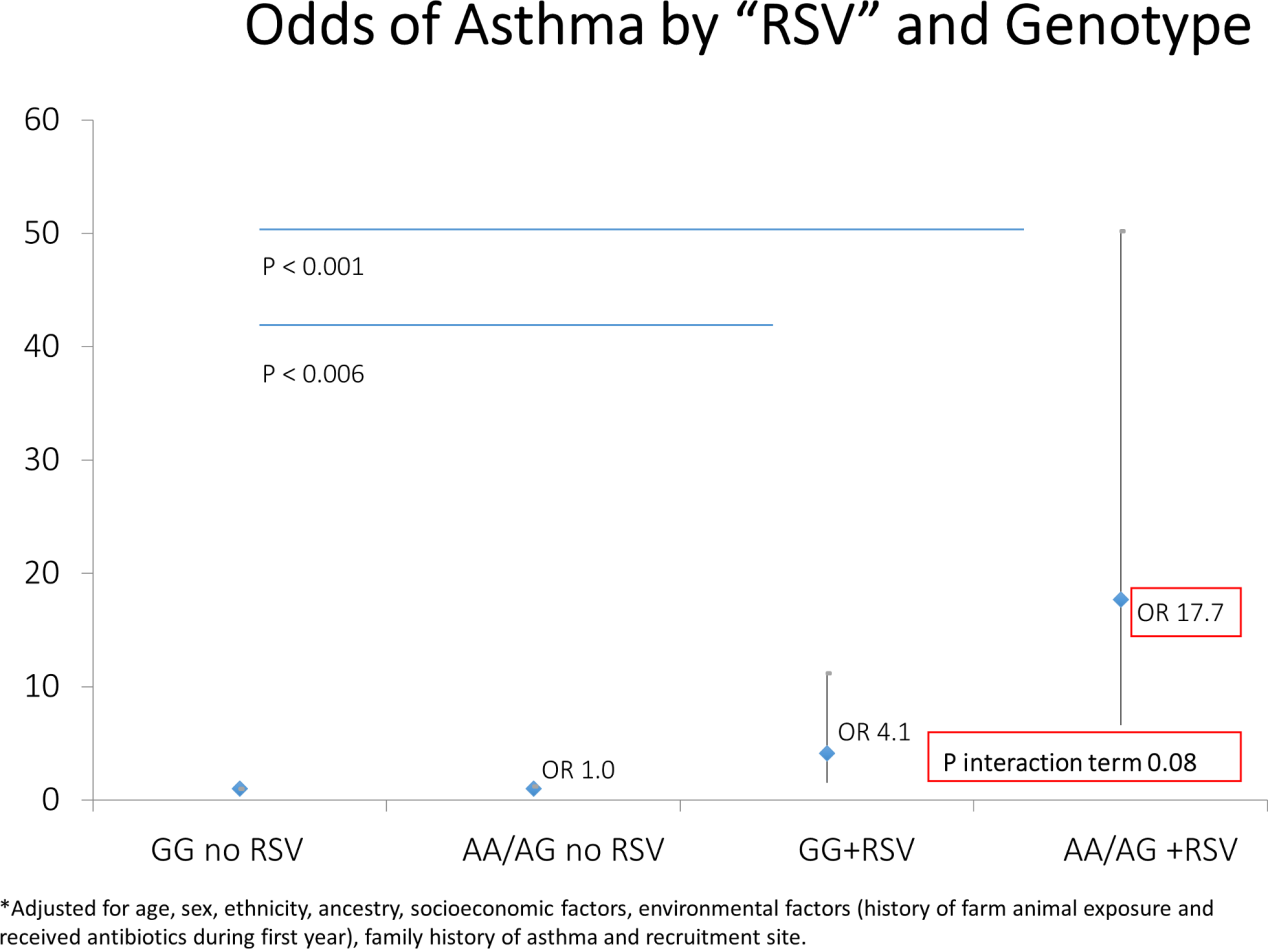
Joint association of bronchiolitis/ RSV and *PAI-1* risk genotype (*rs2227631*) with Asthma. RSV indicates subjects who were diagnosed with bronchiolitis or RSV before age 2 years old. Adjusted for age, sex, ethnicity, ancestry, socioeconomic factors, environmental factors (history of farm animal exposure and received antibiotics during first year), family history of asthma and recruitment site.

**Fig 2:**
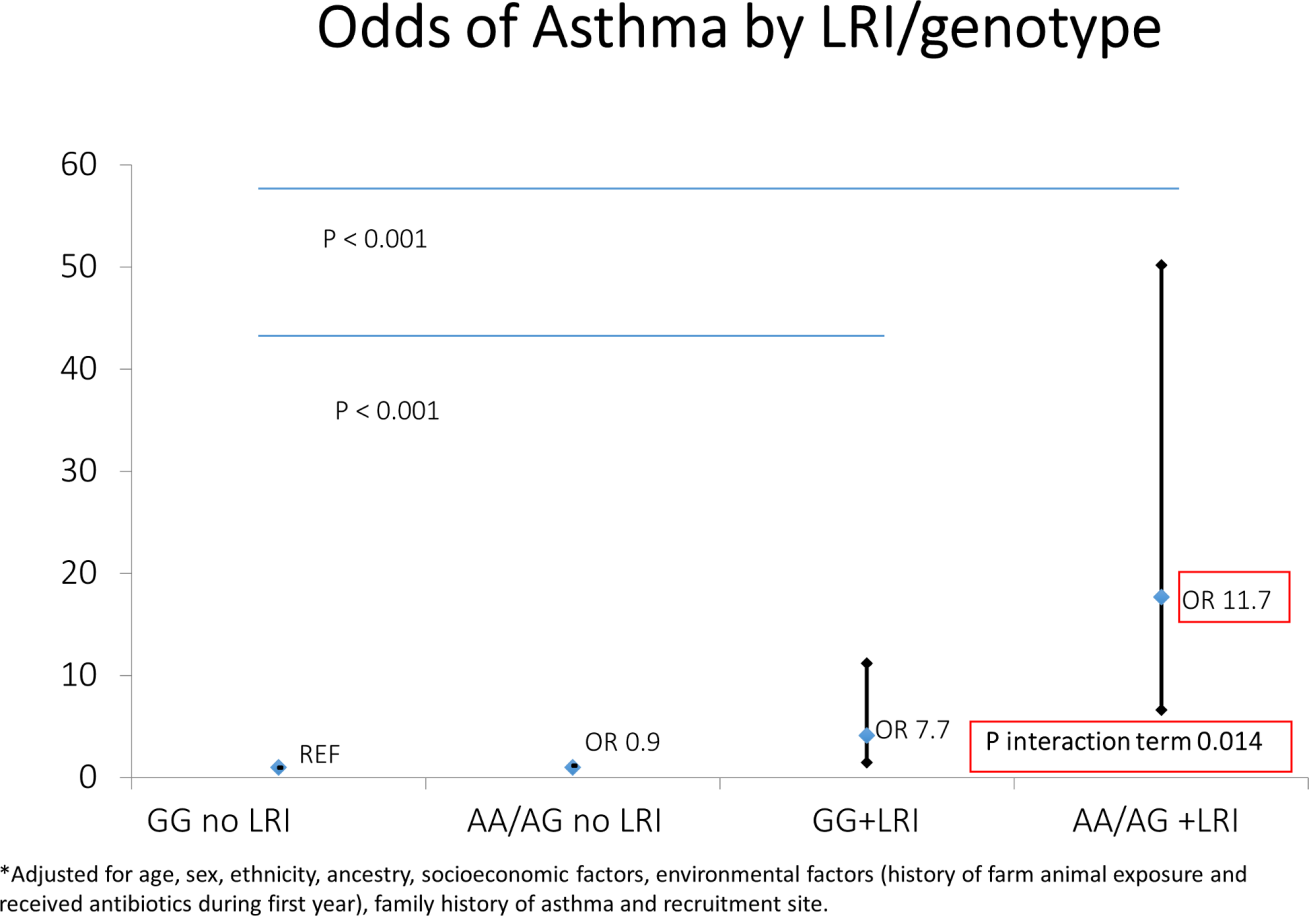
Joint association of early life lower respiratory tract infection and *PAI-1* risk genotype (*rs2227631*) with Asthma. LRI indicates subjects who were diagnosed with a lower respiratory tract infection requiring medical attention before age 2 years old. Adjusted for age, sex, ethnicity, ancestry, socioeconomic factors, environmental factors (history of farm animal exposure and received antibiotics during first year), family history of asthma and recruitment site.

Table 2 presents 2 separate models evaluating the effect of genotype, and infection (tested separately). Firstly, the *PAI-1* SNP *rs2227631* itself does not increase the odds of developing asthma. Second, there was a significant increase in the likelihood of asthma in subjects with a history of early life infection such as RSV bronchiolitis and other LRI (OR 9.9, 95% CI 4.9–20.2; OR 9.1, 95% CI 7.2–11.5, respectively). If the presence of either RSV or LRI was tested in a similar model, the findings were unchanged (OR 10.1, 95% CI 8.2–12.4). In Figs 1 and 2, we display the joint association of the *PAI-1* risk genotype and early life infection with the diagnosis of asthma. While there was an increase of asthma risk in GG-RSV group, there was an even more dramatic increase in the *AG/AA*+RSV group (Fig 1—OR 4.1, 95% CI 1.5–11.2; OR 17.7, 95% CI 6.3–50.2, respectively). Similar findings of lesser magnitude were noted in the *GG*+LRI group and *AG/AA*+LRI group when compared to *GG* no LRI group (Fig 2—OR 7.7, 95% CI 5.5–11.0; OR 11.7, 95% CI 8.8–16.4, respectively). In models testing the interaction terms for LRI-genotype and RSV-genotype (accounting for main effects), the LRI-genotype interaction term was significant at a level of P = 0.014 and the RSV-genotype approached significance at a level of p = 0.08. Furthermore, in a subgroup analysis limited to subjects who had RSV or LRI, there was a clear genotype effect for the risk allele (*AG/AA*+RSV OR 4.3, 95% CI 1.04–17.95; *AG/AA*+LRI OR 1.58, 95% CI 1.06–2.34) compared to those who had RSV or LRI with the wild type *GG* allele. Finally, we also carried out a sensitivity analysis (since RSV was by report) whereby we combined those with either RSV or LRI by these definitions and found that the magnitude of effect was similar for the AA/AG+ either LRI or RSV (OR 12.9, 95% CI 9.6–17.3). The interaction term for any lower respiratory infection and genotype (accounting for main effects) was significant at the P = 0.002 level.

**Table 2:** Adjusted effects of *rs2227631*, and infection on asthma.

Analyses	N	OR^*^ (95% CI)	*P* value^†^
**Genotype alone—*rs2227631***			
*GG*	1405	Reference	
*AG/AA*	2078	1.051 (0.878–1.259)	0.588
**Infection history before age 2 yrs alone**			
RSV bronchiolitis	177	**9.920 (4.881–20.159)**	**0.000**
LRI	1126	**9.110 (7.233–11.474)**	**0.000**

OR, odds ratio; CI, confidence interval; RSV, respiratory syncytial virus

*Adjusted for age, sex, ethnicity, ancestry, socioeconomic factors, environmental factors (history of farm animal exposure and received antibiotics during first year), family history of asthma and recruitment site.

^†^*P* values from multivariate regression analysis. Statistically significant *P* values are indicated in bold.

### Asthma Exacerbations

Table 3 presents 3 separate models (limited to subjects with asthma) evaluating the effect of genotype, infection, and then the joint effect of genotype and infection on asthma severity using three different parameters: steroid use ≥ 2 weeks, ER visit ≥ 2 times, and hospitalization ≥ 2 times all in the previous12 months. Considered the effect of genotype alone, the *AG/AA* genotype showed more than 2 times higher risk of hospitalization (P = 0.046, OR 2.2 95% CI 1.0–4.6). Similarly, subjects with a history of RSV bronchiolitis had significantly higher risk of having an ER visit ≥ 2 times in the previous 12 months (P = 0.001, OR 2.0 CI 95% 1.3–3.1). While findings were similar in direction for LRI, they failed to reach significance (P = 0.18, OR 1.2 95% CI 0.92–1.6, respectively). Finally, the *PAI-1* SNP and RSV jointly increased the risk of ED visits and the risk of hospitalization by 1.8 and 3.1-fold respectively. However, there was no joint effect between the *PAI-1* SNP and LRI on these asthma severity parameters.

**Table 3:** Adjusted effect of *rs2227631*, infection and *rs2227631* gene-infection interaction on asthma exacerbations.

Analyses	Steroid use ≥ 2 weeks in past 12months		ER visit ≥ 2 times in past 12months		Hospitalizations ≥ 2 timesin past 12months	
	OR* (95%CI)	*P*^*†*^	OR* (95%CI)	*P*^*†*^	OR* (95%CI)	*P*^*†*^
**Genotype alone—*rs2227631***						
*GG*	Reference		Reference		Reference	
*AG/AA*	1.078 (0.774–1.501)	0.66	0.931 (0.713–1.216)	0.60	**2.152 (1.013–4.572)**	**0.046**
**Infection history before age 2 yrs alone**						
RSV bronchiolitis	1.243 (0.800–1.931)	0.33	**2.004 (1.314–3.057)**	**0.001**	1.589 (0.740–3.412)	0.23
LRI	1.262 (0.910–1.752)	0.16	1.197 (0.917–1.562)	0.18	1.285 (0.670–2.464)	0.48
**Gene-Infection analyses**						
***rs2227631-RSV***						
*GG* No RSV	Reference		Reference		Reference	
*AG/AA* No RSV	1.089 (0.765–1.550)	0.63	0.911 (0.689–1.204)	0.51	1.728 (0.794–3.761)	0.17
*GG*+RSV	1.255 (0.529–2.977)	0.61	**2.408 (1.053–5.506)**	**0.04**	‡	0.99
*AG/AA*+RSV	1.274 (0.734–2.211)	0.39	**1.854 (1.111–3.092)**	**0.02**	**3.107 (1.143–8.450)**	**0.03**
***rs2227631- LRI***						
*GG* No LRI	Reference		Reference		Reference	
*AG/AA* No LRI	0.954 (0.547–1.664)	0.87	0.709 (0.469–1.069)	0.10	2.075 (0.566–7.602)	0.27
*GG*+LRI	1.115 (0.634–1.961)	0.71	0.859 (0.561–1.315)	0.49	1.195 (0.278–4.970)	0.80
*AG/AA*+ LRI	1.278 (0.758–2.155)	0.36	0.991 (0.668–1.472)	0.96	2.645 (0.750–9.334)	0.13

OR, odds ratio; CI, confidence interval; RSV, respiratory syncytial virus

*Adjusted for age, sex, ethnicity, socioeconomic factors, environmental factors (history of smoke exposure), medication for asthma control and recruitment site. Analyses of SNP and SNP-infection also controlled for ancestry.

^†^*P* values from multivariate regression analysis. Statistically significant *P* values are indicated in bold.

‡ unable to estimate stable odds ratio due to small cell size.

### Lung function

Table 4 presents 3 separate models (limited to subjects with asthma) evaluating the effect of genotype, infection, and then the joint effect of genotype and infection on lung function including three parameters; FEV_1_% predicted, FVC % predicted, and FEV_1_/FVC % predicted. Only the FEV_1_/FVC ratio was reduced in AG/AA genotype compared to GG genotype. Early life history of LRI was associated with decreases in FEV_1_/FVC ratio; but this was not significant for RSV bronchiolitis. When we looked at the joint effect of PAI-1 genotype-and early life infections, FEV_1_% predicted, and FEV_1_/FVC % predicted were further reduced in *AG/AA*+LRI group compared to *GG* no LRI group (P = 0.03, coefficient β -2.06 95% CI -3.97–-0.16; and P = 0.001, coefficient β -1.97 95% CI -3.10–-0.84, respectively). Similar findings were noted in the analyses of RSV with lower FEV_1_% predicted in AG/AA-RSV group. The findings for FEV_1_/FVC % predicted neared significance but showed a similar magnitude and direction of findings.

**Table 4:** Adjusted effect of *rs2227631*, infection and *rs2227631*gene-infection interaction on lung function in asthmatic subjects.

Analyses	FEV_1_% predicted		FVC % predicted		FEV_1_/FVC ratio % predicted	
	Coefficient β* (95% CI)	*P*^*†*^	Coefficient β* (95% CI)	*P*^*†*^	Coefficient β* (95% CI)	*P*^*†*^
**Genotype alone—*rs2227631***						
*GG*	Reference		Reference		Reference	
*AG/AA*	-1.212 (-2.530–0.105)	0.07	-0.517 (-1.812–0.778)	0.43	**-0.787 (-1.570 –-0.003)**	**0.049**
**Infection history before age 2 yrs alone**						
RSV bronchiolitis	-1.854 (-4.315–0.606)	0.14	-1.032 (-3.458–1.393)	0.40	-0.988 (-2.430–0.453)	0.18
LRI	-1.195 (-2.528–0.138)	0.08	-0.060 (-1.256–1.370)	0.93	**-1.311 (-2.090 –-0532)**	**0.001**
**Gene-Infection analyses**						
***rs2227631-RSV***						
*GG* No RSV	Reference		Reference		Reference	
*AG/AA* No RSV	-1.051 (-2.414–0.312)	0.13	-0.299 (-1.639–1.041)	0.66	**-0.844 (-1.655 –-0.338)**	**0.04**
*GG*+RSV	0.303 (-3.794–4.400)	0.88	1.005 (-3.024–5.033)	0.62	-0.848 (-3.285–1.589)	0.49
*AG/AA*+RSV	**-3.113 (-6.050 –-0.177)**	**0.04**	-1.741 (-4.628–1.146)	0.23	-1.601 (-3.347–0.146)	0.07
***rs2227631-LRI***						
*GG* No LRI	Reference		Reference		Reference	
*AG/AA* No LRI	-0.757 (-2.546–1.032)	0.41	-1.000 (-2.759–0.758)	0.26	0.243 (-0.816–1.301)	0.65
*GG*+LRI	-0.326 (-2.382–1.731)	0.75	-0.348 (-2.369–1.673)	0.74	-0.095 (-1.122–1.311)	0.88
*AG/AA*+ LRI	**-2.063 (-3.971 –-0.155)**	**0.03**	-0.234 (-2.109–1.641)	0.81	**-1.971 (-3.101 –-0.843)**	**0.001**

FEV1, forced expiratory volume in 1 second; FVC, forced vital capacity; BD, bronchodilator; CI, confidence interval; RSV, respiratory syncytial virus

*Adjusted for BMI, socioeconomic factors, medication for asthma control and recruitment site. Lung function parameters (% predicted values) were already adjusted with age, sex, and race. Additionally genotype and genotype-infection analyses were corrected for ancestry.

^†^*P* values from multivariate regression analysis. Statistically significant *P* values are indicated in bold.

### Replication

We evaluated the asthma associations in the SAGE cohort with similar results. The SNP itself was not associated with asthma (OR 1.27, 95% CI 0.85–1.88), while both RSV (OR 14.3, 95% CI 1.71–119.53) and LRI (OR 22.1, 95% CI 11.8–41.4) were associated. We also replicated the joint effects for SNP-LRI association. While there was an increase of asthma risk in GG-LRI group, there was an even more dramatic increase in the *AG/AA*+LRI group (OR 20.4, 95% CI 8.9–46.9; OR 26.7, 95% CI 11.2–63.9, respectively). Despite a similar direction and magnitude of association, we were not able to replicate the SNP-RSV associations in the smaller SAGE cohort due to small numbers (n = 9) in this sub-group (OR 5.81, 95%CI 0.54–62.3 for *AA/AG*+RSV group). We also were not able to replicate the exacerbation and lung function associations in SAGE, possibly due to the smaller sample size.

## Discussion

This study examines the effect of a common polymorphism in the *PAI-1* gene and early life lower respiratory infections including RSV/bronchiolitis in patients with asthma. While the genotype itself was not associated with asthma risk, there was a significant interaction between early life infection and genotype on the outcome of asthma diagnosis. Asthma risk increased 17-fold when this genotype was present in individuals with symptomatic RSV / bronchiolitis infection and almost12-fold in those with other LRI in early life requiring medical attention. This finding was replicated in the SAGE II cohort for LRI, but not RSV potentially due to the small numbers of SAGE II subjects in the *AG/AA*+RSV group. Both LRI and RSV infection severe enough to require medical attention (10% and 1% of our control subjects respectively) and the genotype in question are common.[23,24] The frequencies of *AG* and *AA* genotypes were 45.4% and 14.3%; with an overall *A* allele frequency of 37%.

The joint effect of early life lower respiratory tract infection and the gain of function *PAI-1* SNP is in keeping with other studies which suggest that the *PAI-1* pathway is important in airway response to virus, and that an exaggerated response may be detrimental. *PAI-1* plasma levels are increased in young children who had history of URI-induced repeated wheeze.[13] Human rhinovirus infection increases the production of *PAI-1* in primary airway epithelial cells from subjects with asthma, and during an URI in subjects with asthma, nasal lavage and sputum *PAI-1* levels increase.[12] These findings serve as *in vitro* and *in vivo* evidence of impact of respiratory viral infections on *PAI-1* production in asthma. In murine models, *PAI-1* deficiency protected against airway fibrosis, whereas *PAI-1* overexpression enhanced fibrotic changes.[14,15] Blocking *PAI-1* using either siRNA for *PAI-1* or a *PAI-1* inhibitor reduced airway inflammation, tissue remodeling and airway hyperreactivity.[16,17]

The *rs2227631* SNP itself had significant influence on the FEV_1_/FVC ratio within our asthmatic subjects, which is contrast to the findings that this SNP alone was not associated with asthma risk. We did not have sufficient lung function data for non-asthmatic subjects to determine whether there is an effect in normal subjects who would presumably have less airway inflammation and less remodeling potential. Furthermore, early childhood lower respiratory tract infection and the SNP had a joint effect on FEV_1_ and the FEV_1_/FVC ratio. These findings raise the question of whether the *rs2227631* SNP, in the context of viral insult, may cause increased production of airway *PAI-1* enough to affect lung development in infants and cause structural airway changes that lead to lower lung function in subjects with asthma.

This study has a number of limitations. While there was a dose effect for A alleles in our preliminary analyses, our numbers were not sufficiently large to analyze the AA-infection group separately for RSV bronchiolitis. *AG* and *AA* groups were combined for the primary analysis to provide more precise and stable estimates. Secondly, both the PAI-1 SNP *rs2227631* [18,25] and the *4G/5G* polymorphism are promoter site polymorphisms [19,20,26] in strong linkage disequilibrium (D’ = 0.97),[18] making it difficult to determine which is functional even if the *4G/5G* variant had been sequenced. However, others have used the *rs2227631* genotype as a proxy for the *4G/5G* locus, and found it to be the variant which was most highly associated with *PAI-1* levels on GWAS analysis, suggesting that this may be the more important locus.[27] Third, the exposures of RSV/ bronchiolitis and LRI were based on a self-report questionnaire designed to elucidate infections resulting in lower respiratory symptoms in a child under the age of 2 years. This is based on the fact that most childhood respiratory illness is indeed due to viral pathogens as has been shown by the Hartert group.[24] While, it is also possible that a recall bias would have resulted in only more severe illnesses were reported, this is in keeping with our focus. Severe enough illness to require a visit to the physician increase the relevance of inflammation and *PAI-1*. Even if some subjects with less severe illness were systematically included in the “no severe infection group”, this would bias our analysis towards the null hypothesis, making our findings even more robust. Finally, it is also possible that asthmatic subjects may have greater recall of early wheezing illnesses which may increase the magnitude of the wheezing illness–asthma association. However, this effect should not bias the effect of genotype on asthma when studied within these symptomatic subjects as was evaluated in the subgroup analysis. This effect was clearly present, in contrast to the main analysis, which showed no genotype effect. Furthermore, other viruses may have caused symptomatic bronchiolitis and termed “RSV” by health care providers.[24] Over 70% of bronchiolitis is associated with RSV,[24] with most severe bronchiolitis associated with RSV or RSV/rhinovirus co-infection.[28] Thus, RSV is likely to be further enriched in this group beyond 70%. Regardless, our analyses were also consistent for LRI, a proof of the general principle. The importance of any early viral illness in the development of asthma in a susceptible host is underscored by a recent report that all viruses resulting in symptomatic illnesses in the first year of life (not just RV or RSV), increased the risk of asthma by age 7.[29]

## Conclusion

In conclusion, a genetic variant of *PAI-1* which increases *PAI-1* production, together with either early life lower respiratory infection, was associated with asthma diagnosis, asthma exacerbations, and asthma severity based on reduced FEV_1_/FVC ratio in our Latino population. The asthma associations for genotype-LRI were replicated in a smaller African American population. Further prospective studies are needed to replicate our RSV-genotype findings in other non-latino populations, and determine if *PAI-1* variants may serve as a biomarker of risk, which may provide impetus for clinical trials of primary prevention of asthma. In the interim, *PAI-1* genotype in combination with significant LRI, identifies individuals at increased risk of developing asthma. Studies are needed to determine whether interventions affecting airway responses at time of early life LRI in these at risk individuals will decrease the chances of developing asthma.

## Supporting Information

S1 File GALAII supporting data. This file includes data used to perform the primary analyses for this paper.
(TXT)

## Abbreviations

(In order of appearance):

US: United States
PAI-1: Plasminogen Activator Inhibitor-1
ECM: extracellular matrix
SNP: single nucleotide polymorphism
FEV_1_: forced expiratory volume in 1 second
LRI: lower respiratory tract infection
RSV: respiratory syncytial virus
GALA II: Genes-environments and Admixture in Latino Americans
GAGE II: Study of African Americans, Asthma, Genes & Environments II
FVC: Forced Vital Capacity

## References

[1] CDC (2011). Vital Signs.

[2] Fagnani C, Annesi-Maesano I, Brescianini S, D'Ippolito C, Medda E, et al (2008) Heritability and shared genetic effects of asthma and hay fever: an Italian study of young twins. Twin Res Hum Genet 11: 121–131. 10.1375/twin.11.2.121 18361712

[3] Thomsen SF, Kyvik KO, Backer V (2008) Etiological relationships in atopy: a review of twin studies. Twin Res Hum Genet 11: 112–120. 10.1375/twin.11.2.112 18361711

[4] Willemsen G, van Beijsterveldt TC, van Baal CG, Postma D, Boomsma DI (2008) Heritability of self-reported asthma and allergy: a study in adult Dutch twins, siblings and parents. Twin Res Hum Genet 11: 132–142. 10.1375/twin.11.2.132 18361713

[5] Hoffjan S, Nicolae D, Ober C (2003) Association studies for asthma and atopic diseases: a comprehensive review of the literature. Respir Res 4: 14 14748924 10.1186/1465-9921-4-14 PMC314398

[6] Lockett GA, Holloway JW (2013) Genome-wide association studies in asthma; perhaps, the end of the beginning. Curr Opin Allergy Clin Immunol 13: 463–469. 10.1097/ACI.0b013e328364ea5f 23945178

[7] Wjst M, Sargurupremraj M, Arnold M (2013) Genome-wide association studies in asthma: what they really told us about pathogenesis. Curr Opin Allergy Clin Immunol 13: 112–118. 10.1097/ACI.0b013e32835c1674 23222155

[8] Ober C, Vercelli D (2011) Gene-environment interactions in human disease: nuisance or opportunity? Trends Genet 27: 107–115. 10.1016/j.tig.2010.12.004 21216485 PMC3073697

[9] Jackson DJ, Gangnon RE, Evans MD, Roberg KA, Anderson EL, et al (2008) Wheezing rhinovirus illnesses in early life predict asthma development in high-risk children. Am J Respir Crit Care Med 178: 667–672. 10.1164/rccm.200802-309OC 18565953 PMC2556448

[10] Feldman AS, He Y, Moore ML, Hershenson MB, Hartert TV (2014) Toward Primary Prevention of Asthma: Reviewing the Evidence for Early-Life Respiratory Viral Infections as Modifiable Risk Factors to Prevent Childhood Asthma. Am J Respir Crit Care Med. 10.1164/rccm.201405-0901PP PMC4299628 25369458

[11] Caliskan M, Bochkov YA, Kreiner-Moller E, Bonnelykke K, Stein MM, et al (2013) Rhinovirus wheezing illness and genetic risk of childhood-onset asthma. N Engl J Med 368: 1398–1407. 10.1056/NEJMoa1211592 23534543 PMC3755952

[12] Cho SH, Hong SJ, Chen H, Habib A, Cho D, et al (2014) Plasminogen activator inhibitor-1 in sputum and nasal lavage fluids increases in asthmatic patients during common colds. J Allergy Clin Immunol 133: 1465–1467, 1467 e1461-1462. 10.1016/j.jaci.2013.11.009 24373352 PMC4004714

[13] Lee Chung H, Kim SY, Kim SG (2007) Vascular endothelial growth factor and plasminogen activator inhibitor-1 in children with recurrent early wheeze. J Allergy Clin Immunol 119: 1541–1542. 17445877 10.1016/j.jaci.2007.02.040

[14] Eitzman DT, McCoy RD, Zheng X, Fay WP, Shen T, et al (1996) Bleomycin-induced pulmonary fibrosis in transgenic mice that either lack or overexpress the murine plasminogen activator inhibitor-1 gene. J Clin Invest 97: 232–237. 8550840 10.1172/JCI118396 PMC507084

[15] Hattori N, Degen JL, Sisson TH, Liu H, Moore BB, et al (2000) Bleomycin-induced pulmonary fibrosis in fibrinogen-null mice. J Clin Invest 106: 1341–1350. 11104787 10.1172/JCI10531 PMC381464

[16] Lee SH, Eren M, Vaughan DE, Schleimer RP, Cho SH (2012) A plasminogen activator inhibitor-1 inhibitor reduces airway remodeling in a murine model of chronic asthma. Am J Respir Cell Mol Biol 46: 842–846. 10.1165/rcmb.2011-0369OC 22323366 PMC3380292

[17] Miyamoto S, Hattori N, Senoo T, Onari Y, Iwamoto H, et al (2011) Intra-airway administration of small interfering RNA targeting plasminogen activator inhibitor-1 attenuates allergic asthma in mice. Am J Physiol Lung Cell Mol Physiol 301: L908–916. 10.1152/ajplung.00115.2011 21926267

[18] Kathiresan S, Gabriel SB, Yang Q, Lochner AL, Larson MG, et al (2005) Comprehensive survey of common genetic variation at the plasminogen activator inhibitor-1 locus and relations to circulating plasminogen activator inhibitor-1 levels. Circulation 112: 1728–1735. 16172282 10.1161/CIRCULATIONAHA.105.547836

[19] Cho SH, Hall IP, Wheatley A, Dewar J, Abraha D, et al (2001) Possible role of the 4G/5G polymorphism of the plasminogen activator inhibitor 1 gene in the development of asthma. J Allergy Clin Immunol 108: 212–214. 11496236 10.1067/mai.2001.117260

[20] Pampuch A, Kowal K, Bodzenta-Lukaszyk A, Di Castelnuovo A, Chyczewski L, et al (2006) The -675 4G/5G plasminogen activator inhibitor-1 promoter polymorphism in house dust mite-sensitive allergic asthma patients. Allergy 61: 234–238. 16409202 10.1111/j.1398-9995.2005.00948.x

[21] Drake KA, Torgerson DG, Gignoux CR, Galanter JM, Roth LA, et al (2014) A genome-wide association study of bronchodilator response in Latinos implicates rare variants. J Allergy Clin Immunol 133: 370–378. 10.1016/j.jaci.2013.06.043 23992748 PMC3938989

[22] Nishimura KK, Galanter JM, Roth LA, Oh SS, Thakur N, et al (2013) Early-life air pollution and asthma risk in minority children. The GALA II and SAGE II studies. Am J Respir Crit Care Med 188: 309–318. 10.1164/rccm.201302-0264OC 23750510 PMC3778732

[23] Staat MA (2002) Respiratory syncytial virus infections in children. Semin Respir Infect 17: 15–20. 11891515 10.1053/srin.2002.31688

[24] Miller EK, Gebretsadik T, Carroll KN, Dupont WD, Mohamed YA, et al (2013) Viral etiologies of infant bronchiolitis, croup and upper respiratory illness during 4 consecutive years. Pediatr Infect Dis J 32: 950–955. 10.1097/INF.0b013e31829b7e43 23694832 PMC3880140

[25] Su S, Chen S, Zhao J, Huang J, Wang X, et al (2006) Plasminogen activator inhibitor-1 gene: selection of tagging single nucleotide polymorphisms and association with coronary heart disease. Arterioscler Thromb Vasc Biol 26: 948–954. 16424345 10.1161/01.ATV.0000204731.17646.f2

[26] Nie W, Li B, Xiu QY (2012) The -675 4G/5G polymorphism in plasminogen activator inhibitor-1 gene is associated with risk of asthma: a meta-analysis. PLoS One 7: e34385 10.1371/journal.pone.0034385 22479620 PMC3313978

[27] Huang J, Sabater-Lleal M, Asselbergs FW, Tregouet D, Shin SY, et al (2012) Genome-wide association study for circulating levels of PAI-1 provides novel insights into its regulation. Blood 120: 4873–4881. 10.1182/blood-2012-06-436188 22990020 PMC3520624

[28] Hasegawa K, Mansbach JM, Teach SJ, Fisher ES, Hershey D, et al (2014) Multicenter study of viral etiology and relapse in hospitalized children with bronchiolitis. Pediatr Infect Dis J 33: 809–813. 10.1097/INF.0000000000000293 24577039 PMC4145057

[29] Bonnelykke K, Vissing NH, Sevelsted A, Johnston SL, Bisgaard H (2015) Association between respiratory infections in early life and later asthma is independent of virus type. J Allergy Clin Immunol 136: 81–86.e84. 10.1016/j.jaci.2015.02.024 25910716 PMC7112259

